# The beneficial effects of angiotensin-converting enzyme II (ACE2) activator in pulmonary hypertension secondary to left ventricular dysfunction

**DOI:** 10.7150/ijms.48096

**Published:** 2020-09-16

**Authors:** I-Chen Chen, Jao-Yu Lin, Yi-Ching Liu, Chee-Yin Chai, Jwu-Lai Yeh, Jong-Hau Hsu, Bin-Nan Wu, Zen-Kong Dai

**Affiliations:** 1 Department of Pediatrics, Kaohsiung Medical University Hospital, Kaohsiung, Taiwan.; 2 Department of Pediatrics, School of Medicine, College of Medicine, Kaohsiung Medical University, Kaohsiung, Taiwan.; 3 Graduate Institute of Medicine, College of Medicine, Kaohsiung Medical University, Kaohsiung, Taiwan.; 4 Department of Surgery, Kaohsiung Medical University, Kaohsiung, Taiwan.; 5 Department of Pathology, School of Medicine, College of Medicine, Kaohsiung Medical University, Kaohsiung, Taiwan.; 6 Department of Pharmacology, School of Medicine, College of Medicine, Kaohsiung Medical University, Kaohsiung, Taiwan.

**Keywords:** pulmonary hypertension, left ventricular dysfunction, renin-angiotensin system, ACE2 activator, ACE2-Ang-(1-7)-MAS axis

## Abstract

Pulmonary hypertension (PH) is a lethal and rapidly progressing disorder if left untreated, but there is still no definitive therapy. An imbalance between vasoconstriction and vasodilation has been proposed as the mechanism underlying PH. Among the vasomediators of the pulmonary circulation is the renin-angiotensin system (RAS), the involvement of which in the development of PH has been proposed. Within the RAS, angiotensin-converting enzyme 2 (ACE2), which converts angiotensin (Ang) II into Ang-(1-7), is an important regulator of blood pressure, and has been implicated in cardiovascular disease and PH. In this study, we investigated the effects of the ACE2 activator diminazene aceturate (DIZE) on the development of PH secondary to left ventricular dysfunction. A model of PH secondary to left ventricular dysfunction was established in 6-week-old Wistar rats by ascending aortic banding for 42 days. The hemodynamics and pulmonary expression of ACE, Ang II, ACE2, Ang-(1-7), and the Ang-(1-7) MAS receptor were investigated in the early treatment group, which was administered DIZE (15 mg/kg/day) from days 1 to 42, and in the late treatment group, administered DIZE (15 mg/kg/day) from days 29 to 42. Sham-operated rats served as controls. DIZE ameliorated mean pulmonary artery pressure, pulmonary arteriolar remodeling, and plasma brain natriuretic peptide levels, in addition to reversing the overexpression of ACE and up-regulation of both Ang-(1-7) and MAS, in the early and late treatment groups. DIZE has therapeutic potential for preventing the development of PH secondary to left ventricular dysfunction through ACEII activation and the positive feedback of ANG-(1-7) on the MAS receptor. A translational study in humans is needed to substantiate these findings.

## Introduction

Pulmonary hypertension (PH) is a progressive, incurable disorder that complicates the majority of cardiovascular and respiratory diseases 1, 2. According to the 6th World Symposium on Pulmonary Hypertension of 2018, the updated clinical classification includes five types of PH 3. By far the most common form of PH is that due to left heart disease (group II PH), which is associated with high morbidity and a poor prognosis 4, 5. PH is the product of multiple complex and multifactorial processes, but its diagnosis remains challenging. In group II PH, triggered by a retrograde increase in left atrial pressure (LAP), mechanisms including proliferative vasculopathy, vascular remodeling, vascular constriction, and endothelial cell dysfunction have been cited 5-7.

The renin-angiotensin system (RAS) is composed of two opposing arms. The vasopressor arm, or classic RAS, includes angiotensin-converting enzyme (ACE), with angiotensin (Ang) II as the enzymatic product, and the Ang II type 1 (AT1) receptor, which mediates the biological actions of Ang II. The vasodilator arm, or alternative RAS, consists of angiotensin-converting enzyme 2 (ACE2), Ang-(1-7), produced by the hydrolysis of Ang II, and the Ang-(1-7) MAS receptor, which mediates the vasodilatory, antiproliferative, antifibrotic, and antithrombotic effects of Ang-(1-7) 8-13. ACE2 is an important regulator of the RAS and plays an important role in balancing vasoconstriction and vasodilation 11, 14. Recently, the RAS has been implicated in the etiology of PH 8-10. However, whether targeting the ACE2/Ang-(1-7)/MAS receptor pathway is an effective therapeutic strategy is unclear.

Diminazene aceturate (DIZE), an anti-trypanosomal drug approved by the US Food and Drug Administration, was recently shown to exert off-target effects, in that it also activates ACE2 15, 16. The action of DIZE in cardiovascular system is poorly understood. One study revealed intravenous DIZE administration markedly reduced right ventricular systolic pressure in monocrotaline-induced PH rats and increased ACE2 and MAS receptor expression in lung tissue 16. The finding was also confirmed by another study, which showed a positive correlation between DIZE and ACE2 and the induction of Ang (1-7)/Mas axis 17.

While ACE2 as a therapeutic target has been studied in animal models of group 1 PH, 16, 18, 19 whether it is a feasible target in other types of PH is unclear. Thus, in this study we used our previously established rat model of group II PH, in which left heart dysfunction is achieved by ascending aortic banding, to investigate: (i) whether ACE/Ang II is upregulated and the ACE2-Ang (1-7)-MAS receptor axis downregulated in our model and (ii) the potential role of DIZE in preventing or ameliorating PH by activating ACE2 to induce Ang (1-7)/MAS receptor expression in aortic-banded rats.

## Methods

### Rat model of group II PH

All protocols were approved by the Animal Research Committee of Kaohsiung Medical University. A Wistar rat PH model (6 weeks old; weight, 220 g) was created by ascending aortic banding, as described previously 20, 21. Briefly, left parasternal thoracotomy in the fourth intercostal space was performed in rats anesthetized with sodium pentobarbitone (20 mg/kg, i.p.) and ketamine (40 mg/kg, i.m.) and orotracheally ventilated using a rodent respirator (Harvard, South Natick, MA, USA). A sheathed hypodermic needle (19-gauge) was placed along the axis of the ascending aorta, and a length of 3-0 nylon suture was tied around both. The needle was then removed, leaving a stenosis in the ascending aorta 1 cm distal to the aortic valve. Sham-operated rats of similar body weight served as controls and underwent an identical procedure, except that the aorta was not banded. The day of aortic banding was designated as day 0. Effective aortic banding was confirmed by a pressure gradient around 40 mmHg, determined by transthoracic echocardiography (5-MHz transducer; Hewlett-Packard, Palo Alto, CA, USA) on day 1. All animals were housed individually in a 12-h dark/light cycle-controlled room and fed a regular rat chow diet.

According to our previous protocol 20, 21, rats in the early treatment protocol were randomized to undergo sham operation or ascending aortic banding, and aortic-banded rats were further randomized for subcutaneous treatment with saline (AOB_42_) or DIZE at a dose of 15 mg/kg/day, from days 1 to 42 (AOB_42_/DIZE_1-42_) (Supplement, Fig S1). Rats in the late treatment protocol were randomized to undergo sham operation or ascending aortic banding. The rats were then further randomized to receive saline (AOB_42_) or DIZE at 15 mg/kg/day (AOB_42_/DIZE_29-42_) for 14 days, starting on day 29. All of the rats were euthanized on day 42.

### Hemodynamic measurements

A tracheotomy was performed in rats anesthetized with sodium pentobarbitone (20 mg/kg, i.p.) and ketamine (60 mg/kg, i.m.). Blood pressure was recorded as follows: a PE-50 catheter (Becton-Dickinson, Sparks, MD, USA) was inserted into the femoral artery using a cut-down procedure, and a left parasternal thoracotomy was then performed. A catheter was inserted via the left auricle into the left atrium, followed by the insertion of a second catheter into the main pulmonary artery via the right ventricular outflow tract. Pulmonary and femoral artery pressures were recorded simultaneously using a polygraph system (BIOPAC Systems, Inc., Goleta, CA, USA). Further details are provided in our previously published study 21.

### Tissue preparation and histopathologic analysis

After hemodynamic monitoring, the lungs and heart were rapidly perfused with normal saline under a pressure of 100 cm H_2_O prior to their removal. The banded segment of each aorta was dissected and examined under an operating microscope to confirm the effectiveness of the banding procedure. Three pieces of lung tissue from different lobes were excised from each rat and immersed in 10% formalin for 24 h. The tissue was stained with hematoxylin-eosin (H-E) or other stains, and the medial wall thickness of pulmonary arterioles 50 to 100-µm in diameter were evaluated under a microscope at a magnification of 400×. The medial wall thickness of each arteriole was expressed as: percent wall thickness = medial thickness × 2 /external diameter × 100.

One-half of the remaining lung tissue was homogenized for protein extraction, and the other half was frozen in liquid nitrogen and stored at -70°C for western blot analysis. The right ventricle (RV) was isolated by dissection along its septal insertion. The RV, left ventricle (LV), and interventricular septum (S), as well as the lung, were weighed to determine the extent of RV and LV+S hypertrophy.

### Ang II and Ang-(1-7) measurements

Plasma (0.8-1.0 mL) and lung (~100 mg) supernatants were acidified with 0.6% trifluoroacetic acid (TFA) to obtain a 10% (w/v) homogenate. The samples were centrifuged at 2,000 × g for 15 min at 4°C, washed, and dried under a steam of nitrogen at 60°C. Enzyme-immunoassay kits (Phoenix Pharmaceuticals, CA, USA) were used to measure the Ang II and Ang-(1-7) contents of the lung.

### Immunohistochemical staining for pulmonary Ang-(1-7)

Formalin-fixed, paraffin-embedded 5-mm-thick tissue sections were prepared for immunohistochemistry as follows: the sections were deparaffinized, hydrated, and treated with 0.3% hydrogen peroxide in methanol to eliminate endogenous peroxidase activity. They were then washed with phosphate-buffered saline (PBS), incubated with anti-Ang-(1-7) antibody (Oncogene, Boston, MA, USA) for 1 h at room temperature, washed again with PBS and incubated for 30 min with biotinylated second antibody (DAKO, Glostrup, Denmark). After a further PBS wash, the specimens were incubated for 30 min with peroxidase-labeled streptavidin (DAKO), followed by incubation with diaminobenzidine (Sigma, St. Louis, MO, USA) and counterstaining with Mayer's hematoxylin. The sections were then examined under a light microscope.

### Western blotting of ACE, ACE2 and the MAS receptor

Tissue (100 mg) was homogenized in 1 ml of RIPA buffer (1% Triton X-100, 15 mM HEPES-NaOH [pH 7.5], 0.15 mM NaCl, 1% sodium deoxycholate, 0.1% SDS, 1 mM sodium orthovanadate, 10 mM EDTA, and 0.5% protease inhibitor cocktail [Sigma]) and centrifuged at 15,000 × g for 20 min at 4°C. The protein (100 µg) was subjected to SDS-PAGE with a 10% polyacrylamide gel and transferred onto a PVDF membrane (Pall, Port Washington, NY, USA). The membrane was blocked with 5% non-fat dry milk in Tris-buffered saline (TBS), probed with anti-actin (1:10,000) (Upstate Biotechnology, Lake Placid, NY, USA), anti-ACE, anti-ACE2 (1:500 dilution; Millipore, Billerica, MA, USA), or anti-MAS (1:1,000 dilution; Millipore) antibodies, and then incubated with horseradish peroxidase-conjugated secondary antibody. Signals were detected using the Western Lighting® chemiluminescent kit (Millipore) according to the manufacturer's specifications.

### Statistical analysis

The results obtained from the western blots were analyzed by densitometry and are expressed as the mean ± standard error of the mean (SEM). All data were analyzed by one-way analysis of variance (ANOVA) followed by Tukey's test. The beneficial effects of DIZE were compared between the early and late treatment protocols using a two-way ANOVA (n=6-8 rats/group). A *P-*value < 0.05 was considered to indicate statistical significance.

## Results

### DIZE reduces pulmonary arterial pressure, left atrial pressure, LV and RV hypertrophy, and pulmonary vascular remodeling

**Figure 1:**
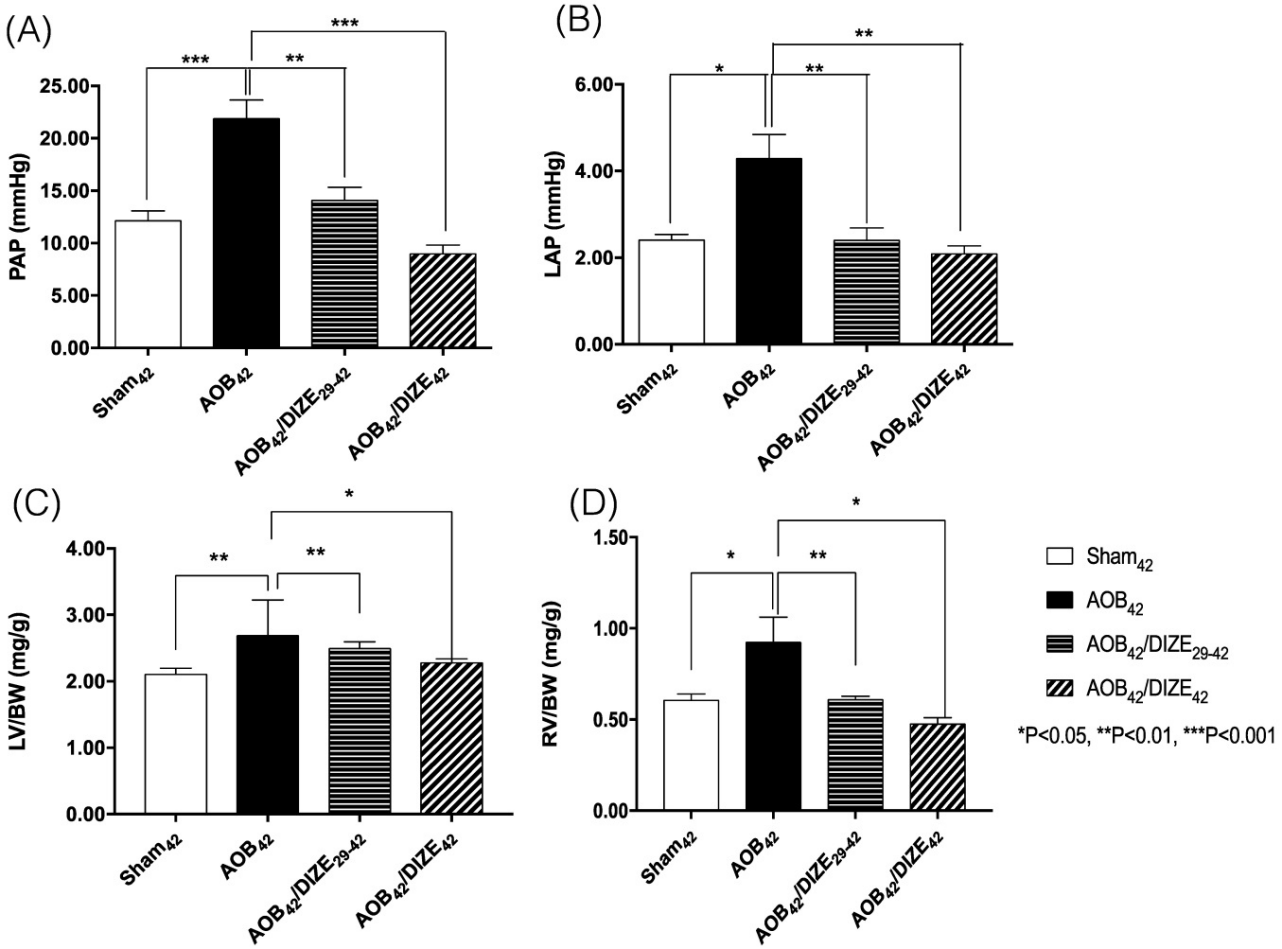
Comparison of (A) pulmonary arterial pressure (PAP), (B) left atrial pressure (LAP), (C) the ratio of left ventricle weight to body weight (LV/BW), and (D) the ratio of right ventricle weight to body weight (RV/BW) among the sham_42_, AOB_42_, AOB_42_/DIZE_29-42_ and AOB_42_/DIZE_42_ groups. PAP and LAP levels were significantly higher in the AOB_42_ group than in sham-operated rats, and significantly lower in in AOB_42_/DIZE_1-42_ and AOB_42_/ST_29-42_ rats than in AOB_42_ rats. Both LV/BW and RV/BW were higher in the AOB_42_ groups than in the sham-operated groups. LV/BW and RV/BW were significantly lower in in AOB_42_/DIZE_1-42_ and AOB_42_/ST_29-42_ groups than in the AOB_42_ groups. Values represent the mean ± SEM. *P<0.05, **P<0.01, ***P<0.001.

Both the mean pulmonary arterial pressure (PAP) (Fig. 1A) and the LAP (Fig. 1B) increased significantly in the AOB_42_ group compared to the sham-operated group. However, decreases in PAP and LAP were observed in both the early (AOB_42_/DIZE_1-42_) and late (AOB_42_/DIZE_29-42_) treatment groups.

The ratios of LV weight to body weight (LV/BW) (Fig. 1C) and RV weight to body weight (RV/BW) (Fig. 1D) were significantly higher in AOB_42_ rats than in sham_42_ rats, which indicated hypertrophy of both ventricles in the AOB_42_ rats. In the early (AOB_42_/DIZE_1-42_) and late (AOB_42_/DIZE_29-42_) treatment groups, both LV/BW and RV/BW decreased in response to DIZE.

The femoral artery pressure was similar among the sham-operated rats, the AOB_42_ group, the early (AOB_42_/DIZE_1-42_), or late (AOB_42_/DIZE_29-42_) treatment groups (Fig. S2).

**Figure 2:**
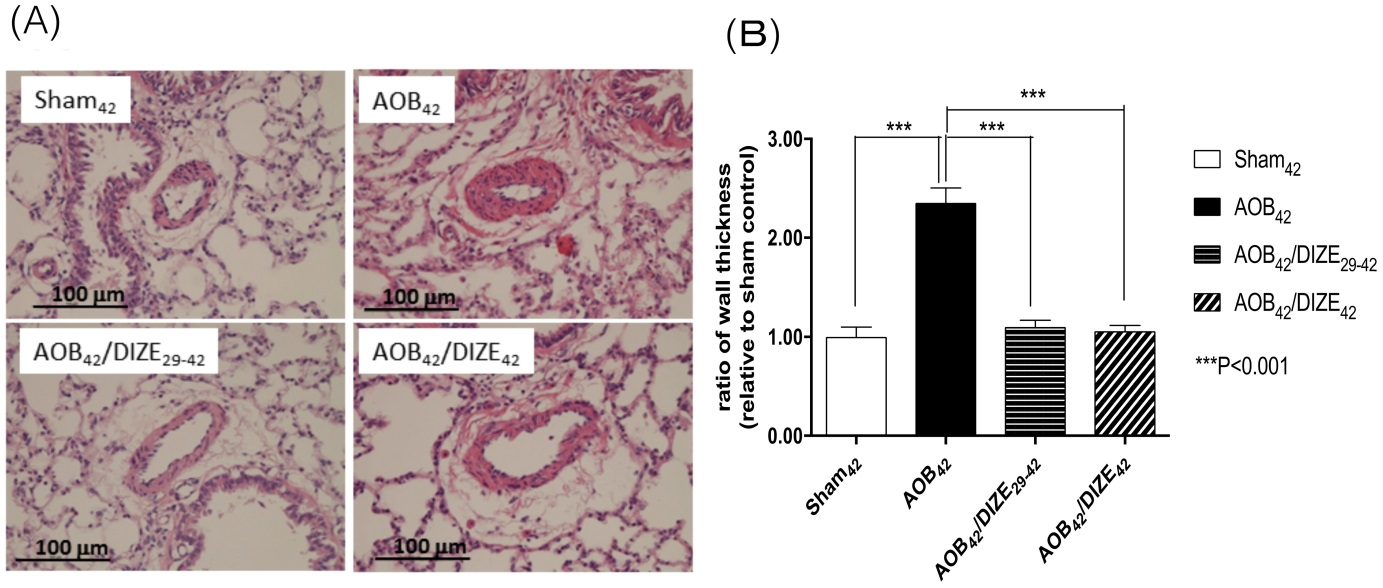
(A) Hematoxylin-eosin staining of lung tissue (magnification, 200×). (B) The muscular layer of pulmonary arterioles 50-100 µm in diameter was significantly thicker in AOB_42_ rats than in sham_42_, AOB_42_/DIZE_1-42_ or AOB_42_/DIZE_29-42_ rats. Values represent the mean ± SEM. ***P<0.001.

The medial wall of pulmonary arterioles 50-100 μm in diameter was thicker in AOB_42_ rats than in sham-operated rats (Fig. 2). Treatment with DIZE significantly reduced medial wall thickness in the pulmonary arterioles of rats in the early and late treatment groups compared to the respective vehicle-treated AOB_42_ rats.

**Figure 3:**
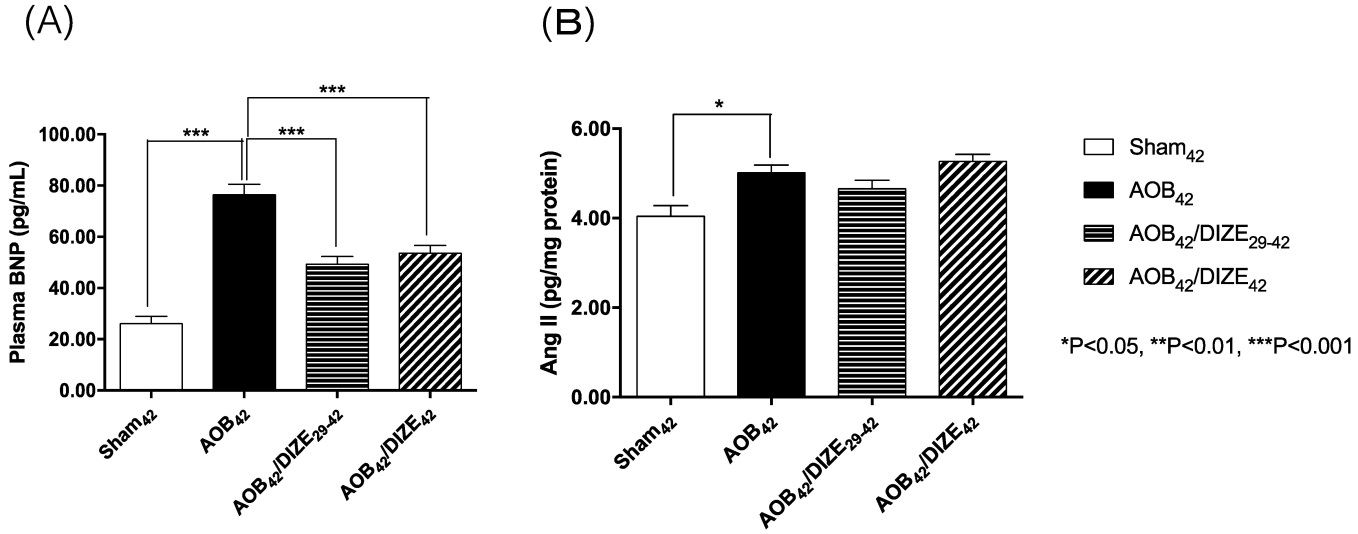
(A) The plasma brain natriuretic peptide (BNP) and (B) pulmonary angiotensin II (Ang II) expression levels. The plasma BNP level was significantly higher in AOB_42_ rats than in sham-operated rats, and significantly lower in AOB_42_/DIZE_1-42_ and AOB_42_/DIZE_29-42_ rats than in AOB_42_ rats. Ang II was significantly higher in AOB_42_ rats than in matched sham_42_ rats, but there was no difference between AOB_42_/DIZE_1-42_ and AOB_42_/DIZE_29-42_ rats versus AOB_42_ rats. Values represent the mean ± SEM. *P<0.05.

The level of plasma brain natriuretic peptide (BNP), a marker of heart failure, was significantly elevated in the AOB_42_ group compared to the matched sham-operated group (Fig. 3A). In both the early (AOB_42_/DIZE_1-42_) and late (AOB_42_/DIZE_29-42_) treatment groups, plasma BNP levels were significantly lower than in the respective vehicle-treated aortic-banded rats.

Ang II, which induces vasoconstriction, was significantly elevated in the AOB_42_ group compared to the matched sham-operated rats. However, neither the early nor the late DIZE treatment protocol altered the level of Ang II in the lung (Fig. 3B).

### DIZE increases pulmonary Ang-(1-7) expression

**Figure 4:**
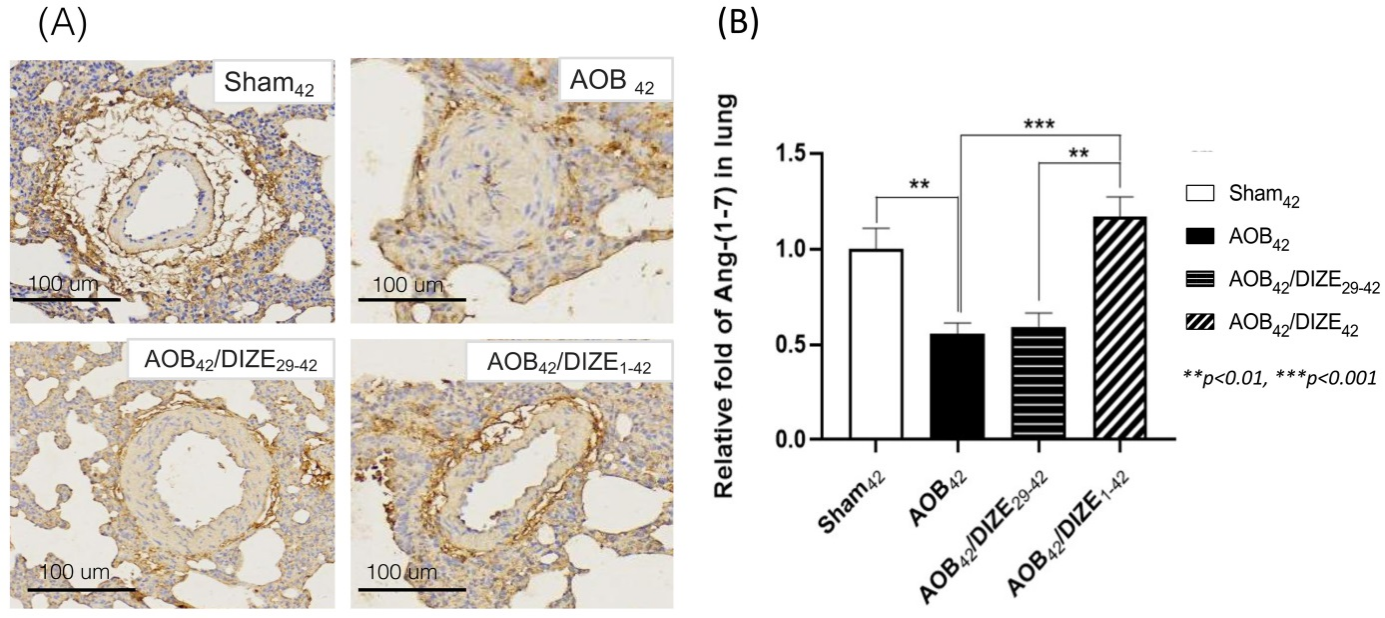
(A) Immunohistochemical staining for Ang-(1-7) in lung tissue (magnification: 200×). (B) Ang-(1-7) expression in lung tissue was significantly lower inAOB_42_ rats than in sham42 rats. DIZE administration increased Ang-(1-7) expression in the AOB_42_/DIZE_1-42_ group, but not in the AOB_42_/DIZE_29-42_ group, compared to the AOB_42_ group. Values represent the mean ± SEM. *P<0.05, ***P<0.001.

Ang-(1-7) opposes the function of Ang II, by inducing vasodilation. In the AOB_42_ group, Ang-(1-7) immunostaining of the pulmonary vasculature was less intense than in sham-operated rats (Fig. 4A). According to the protein analysis, Ang (1-7) expression in the lung was also lower in the AOB_42_ group than in matched sham-operated rats (Fig. 4B), whereas a significant increase in Ang-(1-7) protein expression was observed in AOB_42_/DIZE_1-42_, but not AOB_42_/DIZE_29-42_ rats, compared to the respective vehicle-treated aortic-banded rats (Fig. 4B).

### DIZE increases ACE2 and MAS receptor expression but decreases ACE expression

**Figure 5:**
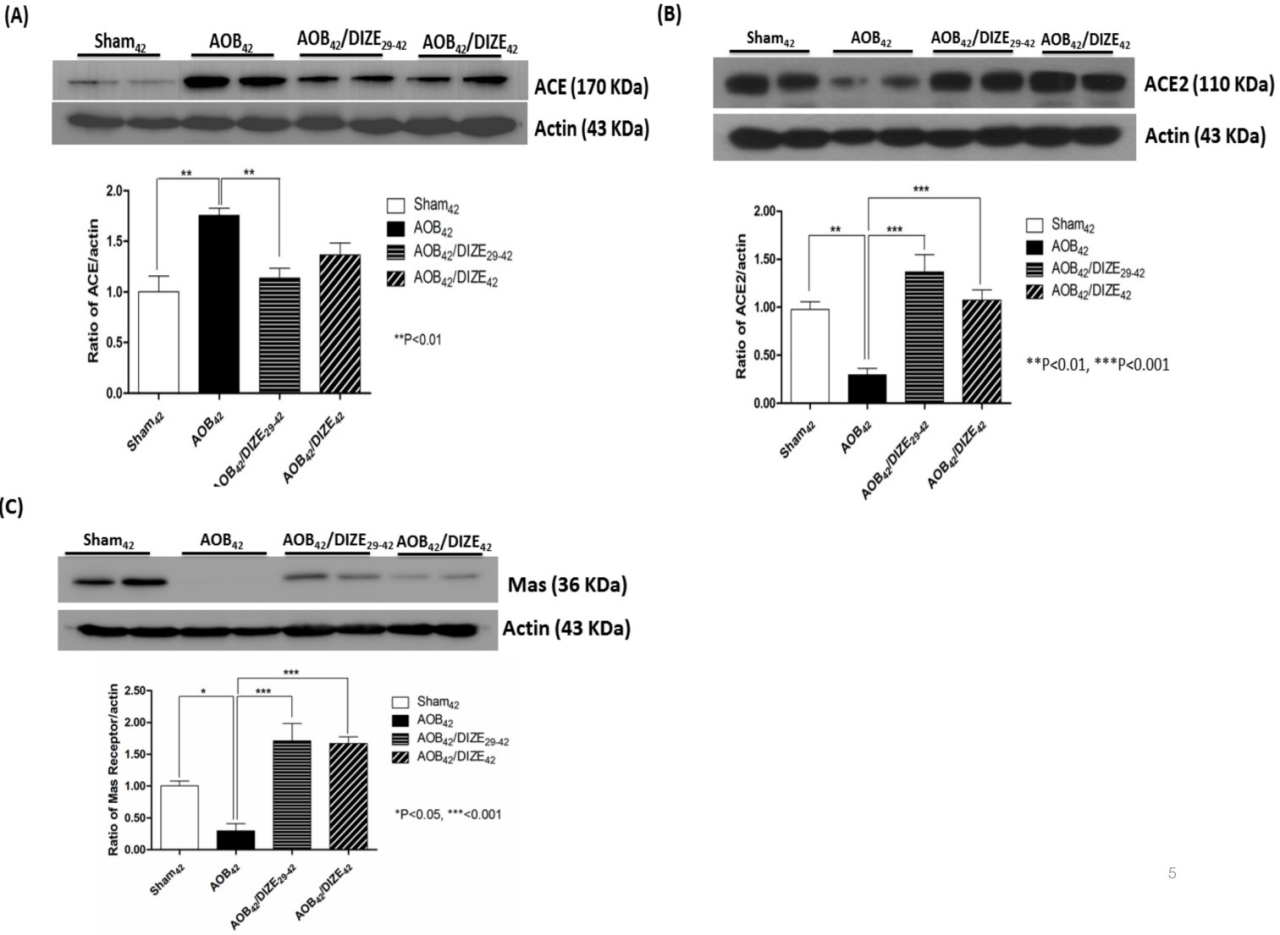
Western blots of ACE, ACE2, and the MAS receptor in the lung of sham_42_, AOB_42,_ AOB_42_/DIZE_1-42_ and AOB_42_/DIZE_29-42_ rats. (A) The left upper panels show the normalized ACE/actin ratios relative to the sham-operated controls. ACE expression was higher in AOB_42_ rats than in sham_42_ rats. DIZE administration decreased ACE expression in AOB_42_/DIZE_29-42_ rats. (B) The right upper panels show the normalized ACE2/actin ratios relative to the sham-operated controls. ACE2 expression was lower in the AOB_42_ group than in the sham_42_, AOB_42_/DIZE_1-42_, and AOB_42_/DIZE_29-42_ groups. (C) The lower panels show the normalized MAS/actin ratios relative to the sham-operated controls. MAS was lower in the AOB_42_ group than in the sham_42_, AOB_42_/DIZE_1-42_, and AOB_42_/DIZE_29-42_ groups. Values represent the mean ± SEM *P<0.05, **P<0.01, ***P<0.001

Aortic banding upregulated pulmonary ACE expression compared to matched sham-operated rats. However, compared to the AOB_42_ group, pulmonary expression of ACE decreased in AOB_42_/DIZE_29-42_ rats, but not in AOB_42_/DIZE_1-42_ rats (Fig. 5A).

DIZE is an ACE2 activator that exerted effects in the rat lung when administered according to our protocol (Fig 5B). ACE2 levels in the lungs of aortic-banded rats were significantly lower than in matched sham-operated rats. In the early treatment (AOB_42_/DIZE_1-42_) and late treatment (AOB_42_/DIZE_29-42_) groups, DIZE significantly increased pulmonary ACE2 levels compared to the respective vehicle-treated aortic-banded rats (Fig. 5B).

MAS, the receptor for Ang-(1-7), contains 325 amino-acid residues and belongs to a family of G-protein-coupled receptors.^22^ Pulmonary MAS expression in our aortic-banded rats was strongly decreased compared to the expression in matched sham-operated rats (Fig. 5C). In the AOB_42_/DIZE_1-42_ and AOB_42_/DIZE_29-42_ groups, MAS receptor levels increased to a similar extent in response to DIZE compared to the respective vehicle-treated aortic-banded rats.

## Discussion

Five types of PH are recognized: group 1, pulmonary arterial hypertension (PAH); group II, PH secondary to left heart disease; group III, pH secondary to lung disease; group IV, PH secondary to chronic pulmonary thromboembolism, and group V, PH with an unclear, multifactorial mechanism 23. PH is characterized by a rise in pulmonary arterial pressure and by pulmonary vascular remodeling, which together result in progressive right heart failure and functional decline. Current PH treatments target pulmonary vascular remodeling and pulmonary vascular tone, based on the molecular pathophysiology of PH, mainly in patients with group I and group IV PH.

However, the largest proportion of PH patients are those with group II PH, the diagnosis of which implies left heart failure 24. In group II PH, LV contraction accounts for > 50% of RV work but it is usually considered as functionally separate from RV contraction 25. A study that enrolled a community cohort of patients with heart failure with a preserved ejection fraction reported a high prevalence rate and more severe course, with the disease not being limited to pulmonary venous hypertension but also including pulmonary arterial hypertension.^26^ In our rat model, aortic banding induces PH by pressure overloading of the LV, thus resembling the systemic hypertension seen in patients with left heart dysfunction. The observed hemodynamic changes, pulmonary arterial remodeling, pathological findings, and protein expression confirm the utility of this model to study group II PH 20, 21.

ACE/Ang II/AT1 constitutes the vasopressor arm of the RAS and the ACE2/Ang-(1-7)/Mas receptor axis counterbalances its potentially harmful effect 13. ACE2 provides negative feedback on the RAS system and protects the heart and kidneys 27. Our study showed that DIZE, an ACE2 activator, also exerts negative feedback on the expression of ACE in the lung 27. In addition, DIZE upregulates the expression of Ang-(1-7) and provides positive feedback on its MAS receptor. The mechanism underlying the positive feedback of Ang-(1-7) on the MAS receptor is unclear but Ang-(1-7) activation of the MAS receptor through the PI3K/Akt/BDNF pathway has been reported 28. Further investigations are needed to clarify the mechanism of action and potential therapeutic value of ACE/Ang1-7/MAS receptor agonists/activators in the treatment of PH.

In our study, Ang II expression in the lung was significantly higher in AOB_42_ rats than in sham-operated rats. However, there was no decrease in the Ang II levels of rats in either the early or the late treatment protocol. However, lung ACE expression was higher in the AOB_42_ group than in the matched sham group, but was normal in the treatment group. This may reflect the very short half-life of Ang II (16 ± 1 s in mice 29), and the fact that it exerts its biological effects by binding to two different receptor subtypes, AT-1 and AT-2. By contrast, ACE is abundant in the small pulmonary arteries and therefore easily detected 30. Thus, measuring ACE instead of Ang II levels in the lung provides a more practical method of assessing hemodynamic changes.

ACE inhibitors and AT_1_R blockers inhibit the ACE/Ang II/AT_1_R axis. While their primary effect is a reduction of systemic blood pressure, they are not effective in patients with PH, who are already at high risk of developing hypotension owing to right ventricular overload 31. The ACE2/Ang (1-7)/MAS receptor axis, responsible for vasodilation in the RAS, may be an important target in the treatment of PH. In a rat model in which monocrotaline was used to induce PAH, beneficial effects, with no obvious adverse impact on blood pressure, were obtained with ACE2 and Ang-(1-7) in the form of a synthetic molecule, and by gene transfer and oral delivery 8, 18, 19, 32-35. Our study, based on a well-established rat model of PH induced by ascending aortic banding, is the first to demonstrate the beneficial effect of targeting ACE2 in group II PH, as demonstrated by the observed hemodynamic changes, pulmonary arterial remodeling, pathological findings, and protein expression.

ACE2 is highly expressed in the lungs, where it converts Ang II to Ang-(1-7) 36. The latter induces vasodilation, antiproliferation, anti-apoptotic and antifibrotic effects by stimulating the MAS receptor, which counterbalances the vasoconstrictive, proliferative, and fibrotic pathways 31. In addition to its relationship with Ang-(1-7), ACE2 also improves endothelial function and suppresses neointimal formation 18, improves the functioning of bone-marrow-derived angiogenic progenitor cells 16, induces apoptosis 12, and suppresses the JAK/STAT and TGF-beta pathways to restore caveolin-1 expression 19.

Human studies showed that serum ACE2 and Ang-(1-7) levels are decreased in patients with PAH related to congenital heart disease, and that mean PAP correlates negatively with serum ACE2 and Ang-(1-7) levels 37, 38. Those results, together with the findings of the present study, suggest that amelioration of PH is related to up-regulation of the Ang-(1-7) receptor MAS in the lung, via ACE2 activation. Our study also demonstrated the protective and therapeutic effects of targeting the ACE2/Ang-(1-7)/MAS receptor pathway in PH, where the expression levels of all three correlated negatively with PAP, LAP, and vascular remodeling in a rat model of group II PH.

## Conclusions

Our animal model of PH secondary to LV dysfunction showed pathological changes and altered protein levels in components of the RAS, including ACE, Ang II, ACE2, Ang-(1-7), and in the MAS receptor. DIZE, as an ACE2 activator, reduced LAP, PAP, and medial wall thickness in pulmonary arterioles, in addition to upregulating pulmonary expression of the ACE2/Ang-(1-7)/Mas receptor axis, leading to preventive and therapeutic effects in a rat model of group II PH. Our study therefore identified DIZE as a novel agent in the treatment of group II PH. Translational studies in humans should be conducted to substantiate these results.

## Supplementary Material

Supplementary figures.

## Abbreviations

PH: pulmonary hypertension
LAP: left atrial pressure
RAS: Renin-angiotensin system
ACE: Angiotensin-converting enzyme
Ang: Angiotensin
ACE2: Angiotensin-converting enzyme 2
DIZE: Diminazene aceturate
AOB: aortic-banded
LV: left ventricle
RV: right ventricle
PAP: mean pulmonary arterial pressure
BW: body weight
BNP: brain natriuretic peptide
PAH: pulmonary arterial hypertension
