# Supplementary material for: The beneficial effects of angiotensin-converting enzyme II (ACE2) activator in pulmonary hypertension secondary to left ventricular dysfunction
Source: Int J Med Sci. 2020 Sep 16;17(16):2594–602. doi: 10.7150/ijms.48096 (PMC7532484; doi:10.7150/ijms.48096)
Supplement: Supplementary file 1 — Supplementary figures. [file ijmsv17p2594s1.pdf]

Supplementary Materials

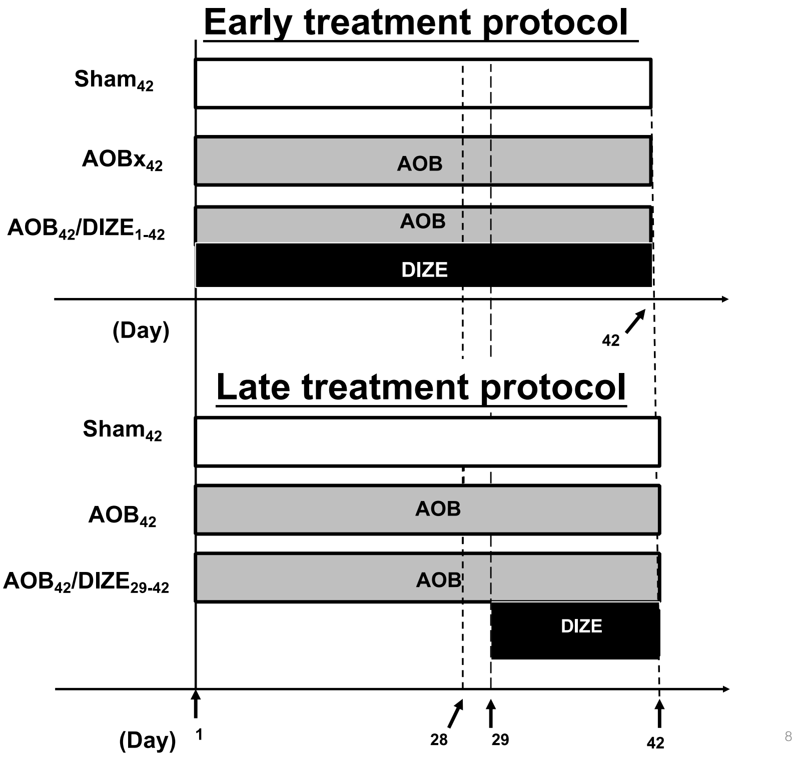

Figure S1. The design of the animal study. The early treatment protocol involved sham-operated rats, ascending aortic-banded vehicle-treated rats (AOB_{42}) and aortic-banded rats treated with 15 mg diminazene aceturate (DIZE)/kg/day from day 1 to day 42 (AOB_{42}/$DIZE_{1-42}$). The treatment protocol involved sham-operated rats ($sham_{42}$), aortic-banded vehicle-treated rats (AOB_{42}), and aortic-banded rats treated with 15 mg DIZE/kg/day from day 29 to day 42 ($AOB4_{42}$/$DIZE_{29-42}$).

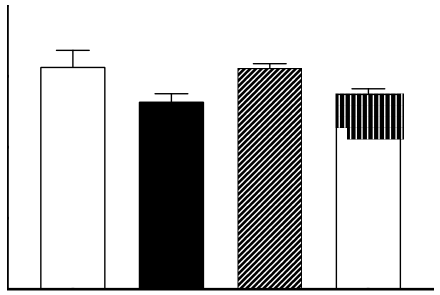

Figure S2. The femoral artery pressures. There is no difference among sham-operated rats, the AOB_{42} group, and the early (AOB_{42}/$DIZE_{1–42}$), or late (AOB_{42}/$DIZE_{29–42}$) treatment groups.
